# Effects of CNS Injury-Induced Immunosuppression on Pulmonary Immunity

**DOI:** 10.3390/life11060576

**Published:** 2021-06-18

**Authors:** Bashir Bietar, Christian Lehmann, Andrew W. Stadnyk

**Affiliations:** Departments of Microbiology and Immunology, Pharmacology, Pediatrics and Pathology, Dalhousie University, Halifax, NS B3H 4R2, Canada; chlehmann@dal.ca (C.L.); andrew.stadnyk@dal.ca (A.W.S.)

**Keywords:** immunosuppression, CNS-injury, stroke, pneumonia, lung

## Abstract

Patients suffering from stroke, traumatic brain injury, or other forms of central nervous system (CNS) injury have an increased risk of nosocomial infections due to CNS injury-induced immunosuppression (CIDS). Immediately after CNS-injury, the response in the brain is pro-inflammatory; however, subsequently, local and systemic immunity is suppressed due to the compensatory release of immunomodulatory neurotransmitters. CIDS makes patients susceptible to contracting infections, among which pneumonia is very common and often lethal. Ventilator-acquired pneumonia has a mortality of 20–50% and poses a significant risk to vulnerable patients such as stroke survivors. The mechanisms involved in CIDS are not well understood. In this review, we consolidate the evidence for cellular processes underlying the pathogenesis of CIDS, the emerging treatments, and speculate further on the immune elements at play.

## 1. Introduction

The main conditions responsible for central nervous system (CNS) injury are trauma, stroke, and spinal cord injuries. Traumatic brain injury (TBI) is one of the leading causes of morbidity and mortality worldwide in individuals under the age of 45 years. Globally, stroke is the second leading cause of death, with 5.5 million deaths per year. Furthermore, stroke results in up to 50% of survivors being chronically disabled [1]. The primary mechanisms of TBI are classified as focal or global (multi-focal) brain damage. Focal brain damage can be caused by injuries of mechanical origin such as car accidents, force from blunt objects, or gunshot wounds. Focal injury pathologies include cerebral lacerations and contusions and various types of hemorrhages. Multi-focal brain damage is due to acceleration/deceleration injuries such as concussions, which result in diffuse axonal injury, ischemic brain injury, or brain swelling [2]. Stroke can be the result of ischemic or hemorrhagic events in the brain. In this review, when referring to stroke it will be in relation to ischemic stroke, which is a focal brain injury due to cerebral blood flow arrest to a particular area of the brain, resulting in rapid local cell death.

The major pathophysiological sequelae of cerebral injury after TBI and stroke is a consequence of direct hypoxic damage to the tissue. Due to terminal membrane depolarization caused by the excessive release of neurotransmitters, voltage-dependent calcium and sodium channels are activated, leading to catabolic intracellular processes mediated by nucleases, lipases, and proteases, which disrupt and degrade membranes and proteins. The upregulation of calcium and sodium levels in ischemic cells stimulate disproportionate reactive oxygen species (ROS) production, which directly damage proteins, lipids, carbohydrates, and nucleic acids [3]. Ultimately, this cascade will result in a complex mix of neuronal and vascular cell death by necrosis, apoptosis, and autophagy. The response of the immune system to these events includes local inflammation governed by the resting microglia. The microglia becomes activated, releasing soluble mediators including pro-inflammatory cytokines and chemokines such as TNF, IL-1ß, IL-6, CCL2-CCL5, and CXCL8 following injury [4]. These mediators upregulate leukocyte adhesion molecules such as intercellular adhesion molecule (ICAM)-1 and P-selectin, and endothelial cell vascular adhesion molecule (VCAM)-1. This ensures that activated leukocytes adhere to endothelial cells and infiltrate injured tissue. In general, the end goal for the local immune response is to eliminate cellular debris and repair salvageable tissue [3].

**Figure 1:**
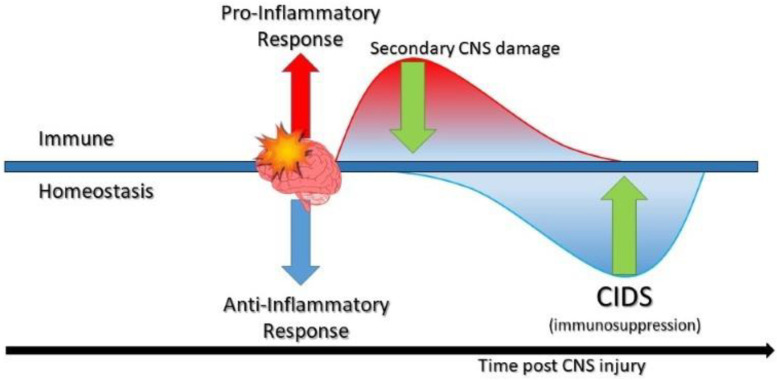
A graphical representation of immune dysregulation following central nervous system (CNS) injury (modified according to Zhou et al., 2019 [8]).

Following, and despite this initial state of local immune activation, the immune response outside the CNS is found to be suppressed and thought to be a major contributor to nosocomial infections that often occur to survivors of CNS injury. The pathophysiology of CNS injury leads to the release of local immunomodulatory factors that activate the sympathetic (SNS) and parasympathetic nervous systems (PNS) and the hypothalamic-pituitary-adrenal axis (HPA). This activation causes the release of a variety of neuromodulators including catecholamines (CA), acetylcholine (AC), and glucocorticoids (GC), which act on the primary and secondary lymphoid organs, altering immune cell function and cytokine production. The consequence is generally decreasing production of pro-inflammatory cytokines while increasing anti-inflammatory cytokines such as IL-10. The net effect is systemic immunosuppression (Figure 1) [5]. Patients in this condition are at higher risk of nosocomial infections. A common infection that affects patients with CNS injury is nosocomial pneumonia. Experimental studies have shown that activated microglia change their morphology from the ramified toward the hypertrophic or amoeboid phenotypes in response to CNS injury. The accumulation of hypertrophic or amoeboid microglia was correlated with neurogenic acute lung injury in rats with neurotrauma. Furthermore, there is evidence that links viral or bacterial lung infection, to activating the microglia in the paraventricular nucleus (PVN) of hypothalamus, which leads to increased hypothalamic IL-6 expression and exacerbated neuroinflammation [6,7]. The predisposition of TBI, stroke, and spinal cord injury patients to pneumonia leads to greater morbidity and increases mortality rates. Understandably, interest has grown in the relationship between this state of immunosuppression and the patients’ response to pneumonia.

This review will compare the healthy lung immune response with the immunosuppressed patient lung immune status and speculate on the mechanisms by which immunosuppression of the lung occurs, and whether this is a point at which CNS injury-induced immunosuppression syndrome (CIDS) may be mitigated.

## 2. Community-Acquired Pneumonia

Community-acquired pneumonia (CAP) is an infection of the pulmonary parenchyma that is acquired outside of the hospital. The condition typically presents with fever, cough, sputum production, chest pain, and difficulty breathing. A chest x-ray is often used to confirm the diagnosis. Pneumonia occurs in about 10% of patients with stroke, especially in the first week after the event, with a three-fold increase in mortality and poor outcome in survivors [9,10]. In comparison, the likelihood to acquire CAP is about 2% in adults ≥65 years old [11]. With this higher incidence in mind, the Pneumonia in Stroke Consensus (PISCES) group defines the spectrum of low respiratory tract infections complicating stroke in the first week, as stroke-associated pneumonia (SAP) [12]. Moreover, there is a strong positive association between age at the time of injury and risk of dying [13]. Pneumonia is a common complication of TBI and occurs in 50–60% of the patients, as TBI patients are prone to aspirate stomach contents [14]. In a study conducted on veterans, it was shown that veterans with spinal cord injuries were more than twice as likely to visit a health care facility for CAP than the general veteran population. CAP was the leading cause of death up to 30 years post-injury, and accounted for 18.9% and 12.7% of deaths in the first and second years, respectively [15]. Other studies reported that pneumonia was the most common cause of death in patients that had survived at least one year following their spinal cord injury [13]. Although most of the cases are hospital acquired, based on the nature of the disease mechanism, CAP is a common enough complication that affects CNS-injured patients that it deserves attention.

Wide-ranging studies on CAP in the pre-penicillin era showed that 95% of the cases were due to *Streptococcus pneumonia* [16]. However, more recent studies applied contemporary FDA-approved diagnostic methods to study the infection in otherwise healthy patients who were hospitalized for CAP and found that a bacterial cause was identified in 27.9%, yet *S. pneumonia* accounted for only 33.3% of the bacterial cases [16]. A similar study conducted by Strålin et al. (2010) showed comparable results, with *S. pneumonia* accounting for 31% of CAP cases, and *Haemophilus influenzae* in roughly 9% and *Mycoplasma pneumoniae* in 12% of cases [17]. The general consensus on CAP is that the most probable etiological agent is bacterial and that *S. pneumoniae* remains the most common, though not the exclusive culprit.

In hospitalized patients with pneumonia after a spinal cord injury, a specific pathogen was identified in 24% of cases and *Pseudomonas aeruginosa* was the etiological agent 21% of the time. CAP caused by *P. aeruginosa* is more common in immunocompromised individuals and has been linked to more severe pneumonia and higher mortality [18]. Overall, the life expectancy of CNS-injured patients is dramatically reduced, and CAP is a large contributor to this poor outcome [19]. Some confounding risk factors of CAP include smoking and alcoholism [20]. The surviving patient must be educated on preventative strategies that lower their risk of pneumonia, by eliminating confounding risk factors when possible.

## 3. Ventilator-Acquired Pneumonia

Ventilator-acquired pneumonia (VAP) refers to pneumonia acquired ≥48 h after endotracheal intubation, with an incidence of 9–27% and 20–50% mortality [21]. The risk of acquiring VAP is significantly increased in TBI patients, with Jovanovic et al., reporting that 49.7% of TBI patients developed VAP. The authors also found a correlation between severity of injury and VAP risk, most likely attributed to time spent on mechanical ventilation. This corresponds with other studies, which have shown an increase of 7% in the chances of contracting VAP for each day spent at the hospital post-TBI [22].

Generally, studies are in agreement that the risk of VAP is around 40–50% in TBI patients, and declare that it is important that this population is well cared for since the impact of TBI is not only acute but have a higher mortality rate 5–20 years post-hospitalization, with both sepsis and pneumonia being more frequent [23]

VAP is often sub-classified as early or late-onset pneumonia. Pneumonia occurring within 4 days of mechanical ventilation is generally caused by antibiotic sensitive bacteria (similar to CAP). Pneumonia that develops more than 4 days after commencing ventilation is often caused by multi-drug resistant (MDR) pathogens, and therefore has a worse outcome.

The diagnosis of VAP represents a clinical challenge. Widely applied clinical criteria for the diagnosis of VAP involve new and persistent radiographic infiltrate, in addition to 2 of the following symptoms: body temperature >38 °C or <36 °C, white blood cell count higher than 12,000 cells/mm^3^ or lower than 4,000 cells/mm^3^, infected tracheal secretions, or gas exchange deficiency [21]. Earlier studies reported MDR pathogens were more commonly associated with late-onset VAP, with the most frequently found MDR pathogens being methicillin-resistant *Staphylococcus aureus* (MRSA), *P. aeruginosa* and other gram-negative bacilli; however, Restrepo et al. (2013) reported that, with the exception of *S. aureus* which was more common in early-onset VAP, pathogens isolated from early-onset vs. late-onset VAP were not significantly different [24]. The finding that bacteria present in the different onsets are similar is also supported by a study by Giantsou et al. (2013), which found that MRSA were the most commonly isolated pathogens in both forms of VAP, at 33% for early and 30% for late VAP, while *P. aeruginosa* isolates were 42% and 47% respectively [25]. In the case of VAP following CNS-injury, *P. aeruginosa*, *S. aureus, Klebsiella pneumoniae*, and *Acinetobacter* species were the most common isolates [26]. Interestingly, Jovanovic et al. also reported that bacterial isolates of CNS-injured-patients with early and late VAP did not differ [27]. These findings favor recent calls to redefine the previously held notion that early and late VAP are two different pathologies.

## 4. Immunocompetent Lung Immunity Against Lower Respiratory Tract Infections

On the backdrop of seemingly common opportunistic respiratory infections in the CNS injured patient population, it is not surprising that the respiratory system is constantly exposed to a variety of microorganisms. Up to the challenge, the lung has a multi-layered system of defense to combat these microorganisms. Innate immunity is coordinated by three major cell types that occupy the airway epithelium: ciliated cells, mucous secreting goblet cells, and the secretory Club cells. These cells enable the epithelium to move inhaled or aspirated bacteria out of the lung through mucociliary transport [28]. In addition, microbes are met with a barrage of anti-microbial molecules, presumably in an effort to achieve a homeostatic balance and not inflammation [29]

Present in the alveolar space, alveolar macrophages are understood to default to an anti-inflammatory role and have a limited capacity to clear bacteria. This is in part a function of the relationship macrophages have with epithelial cells, whereby epithelial cells secrete inhibitory ligands [30]. Additionally, macrophages help induce apoptosis to down-regulate inflammation and therefore regulate the host’s response [31]. On the other hand, and in response to epithelial cell damage, macrophages produce key regulatory inflammatory cytokines, such as IL-1β, which triggers the release of the neutrophil recruiting chemokine, CXCL8, from epithelial cells. In this capacity, the alveolar macrophage can be considered the pivotal decision-maker in whether inflammation is launched.

The burden of removing bacteria that escape mucociliary transport is not borne by macrophages but is the role of recruited neutrophils. In the healthy lung, neutrophils are transiting pulmonary capillaries but not the air spaces [29]. Within hours of infection, neutrophils are recruited to the interstitial compartments and lung air spaces through CXCL8 released by macrophages and epithelial cells [32]. The neutrophils clear the infection through a number of processes including NETosis, phagocytosis, and reactive oxygen species (ROS) production [33].

Neutrophil recruitment is contextual and therefore the response to pneumonia is distinct. Typically, leukocyte recruitment into tissue sites requires transient attachment of adhesion molecules to endothelial cells then subsequently to cells and matrix glycoproteins in the extravascular tissue. Neutrophil recruitment during pneumonia does not follow this pattern and is independent of the selectins [34]. This was tested by Bullard et al. (1996) when they compared neutrophil infiltration elicited by *S. pneumonia* in the lungs of wild type mice and mice deficient in E and P-selectins, expressed on endothelial cells [35]. They found that infiltration 1day post-infection was not impacted by deficiencies in either or both selectins, although bacterial clearance was significantly reduced. Unlike the selectins in this context, integrins are critical to neutrophil migration in response to *Escherichia coli* and *P. aeruginosa*. Blocking antibodies against CD11a and CD11b/CD18 resulted in 80% fewer neutrophils infiltrating compared to mice given a control antibody [36], showing that integrins play an important role for neutrophil infiltration into the infected lung.

Epithelial cell-dependent neutrophil recruitment during pneumonia has focused much research on the ability of the respiratory epithelium to respond to bacteria through pattern recognition receptors (PRRs), such as Toll-like receptors (TLR). The respiratory epithelium possesses most TLRs (10 in humans, 12 in mice). Shimizu et al. (2005) and Cao et al. (2007) showed that TLR2 as well as TLR1 and TLR 6 recognize bacterial peptidoglycans and lipoproteins, and appear to be crucial for the host’s master inflammatory transcriptional regulator, NF-κB, response to both extracellular and intracellular bacteria [37,38]. As receptors for the detection of bacterial products, TLR ought to be the focus of immune therapy in cases of immunosuppression of innate immunity.

Regarding the adaptive immune response in the immunocompetent lung, CD4+ T-cells differentiate from naïve precursors into several possible subsets depending on concurrent cytokine co-stimulation. Upon stimulation through the T-cell antigen receptor from a class II antigen presenting cell, along with co-stimulation by cytokines, T-cells can differentiate into Th1 (develop under interferon (IFN)-γ), Th2 cells (when co-stimulated by IL-4) and Th17 (stimulated by transforming growth factor-β plus IL-6). In turn, these T-cells secrete hallmark effector cytokines. Th1 cells secrete interferon-γ, which plays a critical role in curtailing the growth of intracellular pathogens. Interferon-γ can increase the microbiocidal activity of macrophages by increasing both ROS and reactive nitrogen intermediates. Additionally, Tudhope et al. (2007) showed that interferon receptor signaling resulted in the expression of the angiostatic chemokines CXCL9, CXCL10 and CXCL11, all ligands for CXCR3 expressed on Th1 cells, and through these chemokines Th1 cells promote Th1 recruitment [39]. Th2 cells mediate classical allergic responses, mainly through the secretion of IL-4. Th17 produce IL-17A. Th17 cells are critical in regulating chemokine and anti-microbial protein expression in the lung; patients who fail to develop antigen-specific Th17 cells in response to *S. aureus* develop pulmonary infections [28]. Th17 cells are also essential for mucosal immunity against extracellular pathogens. In summary, Th1 and Th17 cells play important roles in the respiratory adaptive immune response. A significant challenge to bacterial infections is the development of biofilms, especially within the tracheal tube in VAP. The ventilator tube hinders the normal protective upper airway reflexes and inhibits beneficial coughing, resulting in colonization of the oropharynx by aerobic gram-negative bacteria such as *P. aeruginosa*. Additionally, micro aspiration of gastric secretions may contribute to these bacteria becoming established. The bacteria slowly gain access to the lower airway by ventilator cycling and in a state of CIDS, pneumonia risk is increased [40].

## 5. Immunosuppressed Lung: Points of Disruption

Despite our understanding of innate and adaptive immune responses in defense of the respiratory tract, a profound state of immunosuppression is still possible. CNS injuries are linked to immunosuppression and respiratory infections, yet few experimental studies have examined pulmonary immunity in the context of CIDS. Early studies of the immunosuppressed lung were conducted using chemotherapy in mice. In one example, Pennington and Ehrie (1978) showed that the lungs of animals with chemotherapy-induced immunosuppression and subsequently infected with *P. aeruginosa*, were unable to respond with leukocytosis and had a 100% mortality rate. Microscopic observations of *P. aeruginosa*-infected lungs in immunocompetent and immunosuppressed animals revealed significant differences between the groups. Thirty-six hours after infection, increased numbers of alveolar and interstitial macrophages were observed in infected immunocompetent lungs but not in the immunosuppressed lungs. Clearance of *P. aeruginosa* from lung tissue after challenge was 70% by 2 h in immunocompetent animals but significantly less, at 45% at 2 h, in immunosuppressed animals [41]. This example illustrates that infiltration is critical to lung immunity and when there is no leukocytosis there is no infiltration. CNS injuries, on the other hand, do not present with a failure to mobilize cells from the bone marrow. Brommer et al. (2016) modeled CIDS through spinal cord injury (SCI) and showed that only 35% of SCI animals were able to clear a *S. pneumonia* infection from their lungs within 2 days while 86% of sham-injured control animals cleared the infection in the same period of time [42]. Unlike the chemotherapeutic-suppressed animals, CIDS animals did not have a reduction in the numbers of circulating monocytes or macrophages and in fact, the numbers increased, yet immune function was impaired [43].

Doran et al., (2020) showed that immune function was indeed impaired specifically after CNS injury, in a model of TBI, where they found that IL-1β, TNF and ROS levels were significantly diminished in monocytes of TBI mice infected with *S. pneumonia* [44]. In addition to an impaired pro-inflammatory and ROS response, MHC class II was downregulated in alveolar macrophages following a stroke [45]. The failure to present antigens caused by the diminishment of MHC, along with reduced pro-inflammatory cytokines, would presumably cause a failure in crucial neutrophil recruitment. In addition to reduced neutrophil recruitment, there was a decrease in the capacity of the circulating neutrophils to phagocytose pathogens. Furthermore, the ability of these neutrophils to generate an adequate ROS response was also significantly impaired 18–72 h post-trauma, with one study reporting a reduction as high as 40% in the oxidative burst [46]. The impairment of crucial innate elements such as neutrophils and monocytes, likely lead to a significant decrease in bactericidal efficiency and impaired clearing of the lung of bacteria, which leads to pneumonia.

The leukocyte response has been one axis in the cellular events targeted for therapy. In a study investigating the repurposing of tranexamic acid, a plasminogen inhibitor, to treat CIDS following TBI, the drug was able to induce an increase in the numbers of monocytes and macrophages, NK cells and CD4+ and CD8+ T-cells in the blood. Plasmin, derived from proteolysis of plasminogen, is an effector protease in the fibrinolytic system. Apart from a role in the coagulation system, plasmin has been linked to modulation of the immune response by binding to antigen presenting cells and impeding migration into the draining lymph nodes, in effect, suppressing a response [47,48]. Interestingly, despite treatment with tranexamic acid, the bacterial load in the lungs of TBI mice infected with *S. pneumonia* was unaffected. This further favors a theory of impaired immune function rather than an issue of a lack of leukocytosis in the immunosuppressed state related to TBI.

The recruitment and activities of leukocytes are regulated by cytokines. Muehlstedt et al. showed an increase in CXCL8 in the lungs and blood of TBI patients immediately after injury (2 h), reaching maximal levels by 12 h. Concurrently, levels of IL-10 increased both in the lung and systemically; however, unlike CXCL8, IL-10 levels continued to increase well past 60 h post injury, with alveolar levels greater than in the blood. In TBI patients that developed pneumonia, alveolar CXCL8 levels were increased at 12-, 36-, and 60 h post-injury. Again, paralleling CXCL8 levels, alveolar IL-10 concentrations were also significantly higher in the same group. Bronchial lavage IL-10 levels increased from in pneumonia patients but decreased in patients without pneumonia. Alveolar levels of IL-10 and alveolar macrophage HLA-DR expression were inversely correlated within each group [49,50]. At 60 h post-injury, the alveolar macrophage HLA-DR expression of pneumonia patients was significantly less than patients without pneumonia [49,50]. The direct downstream effects of IL-10 are known to be anti-inflammatory, including potent suppression of antigen presentation and cytokine production in APCs, with the consequence of preventing the polarized differentiation of Th1 cells.

**Figure 2:**
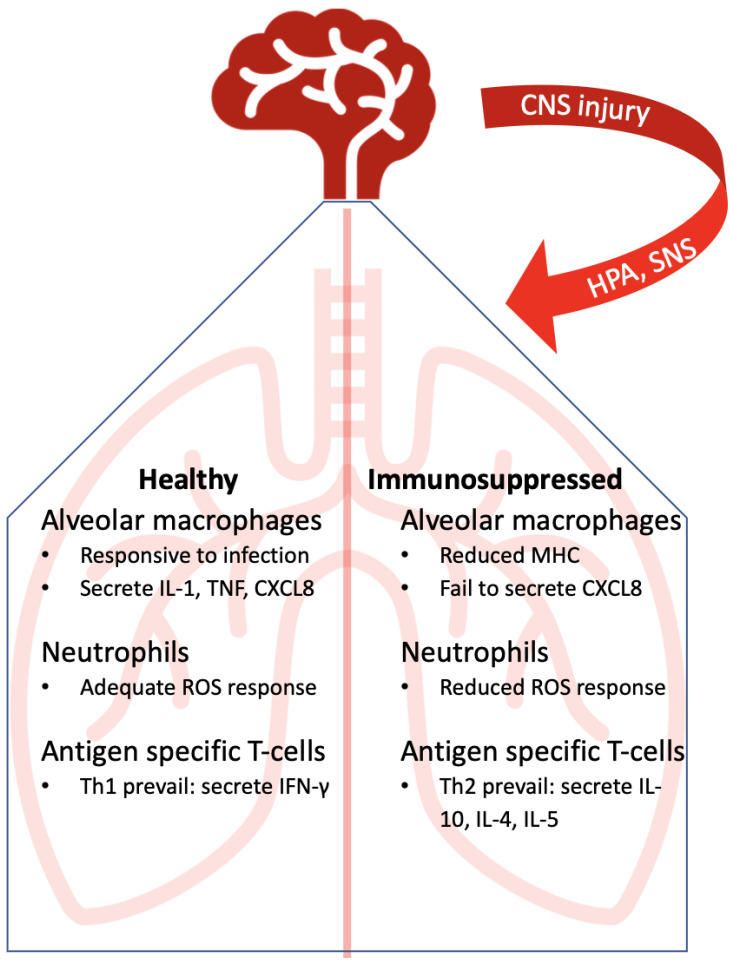
Consequences of CNS injury on the lung.

Further in regard to CNS injury and suppression of adaptive immunity, Członkowska et al. (1979) found that patients with acute cerebral vascular disease had lower blood T-cell counts, with decreased lymphocyte blastogenesis [51]. Haeusler et al. (2008) reported that poststroke patients had an early depression of lymphocyte counts and deactivation of monocytes and Th1 cells (evidenced by declining IFN-γ levels) [52]. These events have been recapitulated in animal studies. Prass et al. (2003) described, in a model of stroke in which the animals also develop pneumonia, that spleen lymphocytes were apoptotic, affecting all lymphocyte subsets, along with a shift from IFN-γ producing Th1 cells to IL-4 and IL-10 producing Th2 cells [53]. These authors also restored protection from the bacterial pneumonia in the ischemic mice through the adoptive transfer of T-cells from healthy mice. This protection was dependent on IFN-γ [53]. No doubt that a shift to Th2 cells will increase IL-10 production and further feeds the immunosuppressive cycle, exacerbating the downstream consequences. Recently, Farris et al. confirmed this immunosuppression state, reporting a decrease in the levels of IL-1β, TNF, IFN-γ, and IL-17A in the lungs of animals with stroke compared to control animals (Figure 2) [54].

Considering that the leukocyte response is important for effective immunity in the respiratory tract, important insights can be gained by studying the consequences to interrupting known or presumed cellular processes on the path to immunosuppression. One recent example used the glucocorticoid receptor blocker, mifepristone in TBI. Mracsko et al. (2014) were able to normalize splenic lymphocyte counts and prevent lymphopenia in mice subjected to CIDS [55]. In addition, β2 adrenergic receptor blockade using ICI 118551 restored T-cell function and restored production of IFN-γ. Römer et al. (2015) blocked both the sympathetic nervous system and hypothalamic–pituitary–adrenal axis using propranolol and mifepristone, in mice subjected to CIDS and pulmonary infection. The outcome was lower infarctions, lower pulmonary infection rates, and increased survival rates in the treated mice [56]. The observation period of their experiment lasted 30 days, during which 73% of treated mice and 52% of untreated mice survived. These findings point to the stress axes as being involved in the immunosuppression.

Another hypothesized element of the immunosuppressive process in CIDS is the upregulation of programmed cell death 1 (PD-1) receptors on resident microglia, infiltrating macrophages, T-cells, B-cells, and antigen presenting cells following CNS injury (presumed to have a neuroprotective role) [57]. The PD-1 ligand, PD-L1, is involved in driving macrophages towards a regulatory role in which cytokine secretion is predominated by anti-inflammatory molecules and decreased production of pro-inflammatory cytokines [58] Furthermore, when PD-1 is bound and activated by either ligand, PD-L1 or PD-L2, it leads to the inhibition of T-cell activation and proliferation. Using an anti-PD-1 antibody to block PD-1 would presumably prevent the suppression, leaving T-cells to respond to any infection that might follow. Ruggeri et al. (2019) used this concept and treated mice deliberately manipulated to model CIDS, with anti-PD-1 antibody. When a low dose of anti-PD-1 antibody was given, significantly more mice survived compared to the control CIDS mice. A closer look at the treatment effects showed that CD4+ T-cells and IFN-γ secreting NKT cells increased in anti-PD-1-treated mice, implicating PD-1 in CIDS.

Increased gut permeability is one possible outcome to systemic immunosuppression and a theory around the gut microbiota and CIDS was triggered by studies that have reported changes in the gut permeability, motility and microbial composition of stroke patients [44,59,60]. The dysbiosis caused by stroke may in fact be a confounding factor in the dysregulated response against *P. aeruginosa* pneumonia. Wang et al., showed a protective role for the microbiota in combating respiratory *P. aeruginosa,* mediated by a theorized microbiotic regulation of neutrophil recruitment and function [61]. Levels of IL-6 and TNF produced against *P. aeruginosa* pneumonia were significantly decreased in antibiotic treated mice modelled to be in a state of dysbiosis compared to “non-dysbiosed/normal” *P. aeruginosa* infected mice. IL-10 levels were also shown to be significantly increased in the antibiotic treated mouse group compared to the non-treated infected mice. Additionally, dysbiosis caused a significant decrease in the expression of the neutrophil chemoattractants CXCL1 and CXCL2. In line with these findings was the novel observation of significantly impaired pulmonary γδ Th17 cells. The γδ Th17 cells are heavily involved in the innate response against pathogens via the production of IL-17A and other pro inflammatory cytokines that switch neutrophil migratory patterns between resting and activated states [61,62]. The discovery of a novel cell-type, in this case γδ T-cells, impacting the reactive capacity in the lung only serves to remind us that there is much to be learned yet about the cells involved in both the protective and immunosuppressive states.

**Figure 3:**
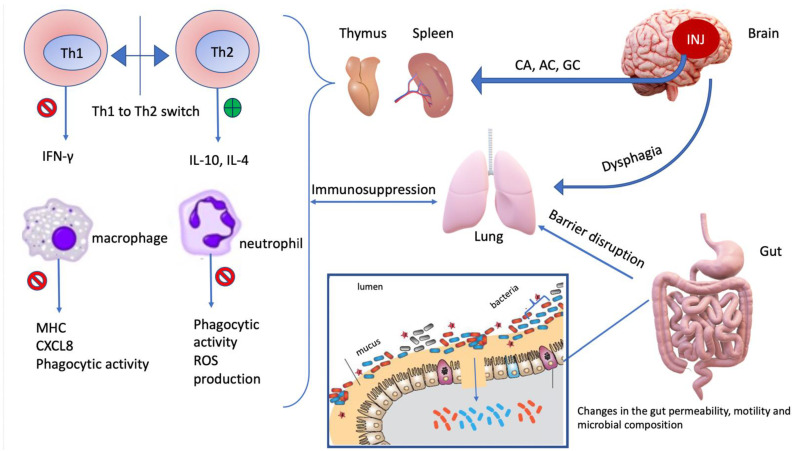
Summary of the immunosuppressive pathology following the initial insult to the CNS that is responsible for higher risk of infection and worsening of patient outcome.

In summary, the cause of CIDS cannot be attributed to a singular event and studies indicate that the immunosuppressive state is multifaceted and incompletely understood. The condition can be described as a challenge to the competence of the immune system in combatting infections on many seemingly disconcerted levels. In the case of CNS injuries, the triggering event is the neuroinflammation caused by the initial insult to the CNS. The downstream consequences of that event can be appreciated to interact even with the gut microbiota (Figure 3). CIDS includes diminished levels of pro-inflammatory cytokines (noteworthy, IFN-γ, TNF and IL-17A) and higher levels of anti-inflammatory cytokines (IL-10), caused by lymphopenia, a Th1/Th2 shift, along with impaired monocyte and neutrophil function and dysregulation of a possibly immunoprotective microbiota.

There is a body of evidence showing that CIDS is a major contributing factor to the development of nosocomial infections, especially pneumonia, and therefore is a concerning sequela of CNS injuries. We have, in this review, demonstrated different points of homeostatic disruptions that can form the basis of research into the pathology of CIDS and multiple potential therapeutic targets.

## 6. Conclusions

The immune system is suppressed in individuals that experience CNS injury, which predisposes them to nosocomial infections. CIDS remains incompletely understood but research on the mechanisms involved, preventative care and immunotherapy is ongoing. Overall, the data support a hypothesis that CNS injury creates an immunosuppressive state, which is different from other immunosuppressive conditions, with specific consequences for the immune response in the lung. Table 1 provides a summary of the events of CIDS detailed above.

## Figures and Tables

**Table 1:** Summary of immune cell specific consequences of central nervous system injury-induced immunosuppression.

Cell Type	Readout	Outcome
Macrophages	Recruitment	↑
	Antigen presentation	↓
	Respiratory burst	↓
	Pro-inflammatory cytokines	↓
Neutrophils	Recruitment	↑
	Respiratory burst	↓
	Phagocytosis	↓
T-cells	Counts	↓
	IFN-gamma	↓
	Th1	↓
	Il-4	↑
	Th2	↑
	Pro-inflammatory cytokines	↓
	Apoptosis	↑

## Data Availability

Not applicable.
